# Feasibility of Enucleation in Standing Sedated Horses With Retrobulbar Anesthesia and Auriculopalpebral Nerve Block: Impact of Ultrasound Guidance in a Case Series

**DOI:** 10.1111/vop.70282

**Published:** 2026-10-27

**Authors:** Simona Lieberth, Katharina Thieme, Christian Dancker, Roswitha Merle, Johanna Corinna Eule

**Affiliations:** ^1^ Unit for Ophthalmology, Centre for Veterinary Clinical Services Freie Universität Berlin Berlin Germany; ^2^ Equine Clinic, School of Veterinary Medicine, Veterinary Hospital Freie Universität Berlin Berlin Germany; ^3^ Pferdeklinik Seeburg Dallgow‐Döberitz Germany; ^4^ Valera Small Animal Clinic Berlin Germany; ^5^ Institute of Veterinary Epidemiology and Biostatistics, School of Veterinary Medicine Freie Universität Berlin Berlin Germany

**Keywords:** analgesia, equine, nerve block, ophthalmology, orbit, surgery

## Abstract

**Objective:**

Examining the feasibility of enucleation in standing sedated horses with dorsal retrobulbar anesthesia (RBA) and auriculopalpebral (AP) nerve block and the impact of ultrasound (US) guidance on RBA success.

**Animals Studied:**

Thirty horses.

**Procedures:**

In a prospective clinical case series, horses (*n* = 15/group) received standardized sedation, dorsal RBA (12 mL mepivacaine) and AP nerve block (4 mL lidocaine). Group 1 received US‐assisted RBA (10 MHz linear probe), Group 2 underwent blind RBA subsequently. Needle positioning time was recorded. Eyelid sensitivity was measured before (*t*_0_) and 20 min after injection (*t*_20_). RBA success was defined as surgical tolerance at *t*_20_. US image quality (excellent/good/poor) was assessed and compared with RBA success. If RBA failed, additional local anesthetic was injected.

**Results:**

RBA success was 63.4% (19/30) with 73.3% (11/15) in Group 1 and 53.3% (8/15) in Group 2 (*p* = 0.179). 3/19 successful cases required additional retrobulbar anesthetic top ups intraoperatively (*t*_60_–*t*_80_). A significant association was found between the number of successfully anesthetized lid areas per horse and RBA success (*p* < 0.001). Mean time for US‐assisted RBA was 5.2 min (±1.7 min). US image quality was rated as “excellent” in 3/15, “good” in 7/15, and “poor” in 5/15 cases, with corresponding success rates of 100% (3/3), 85.7% (6/7), and 40% (2/5), respectively. Improved US image quality showed a non‐significant trend toward higher success (*p* = 0.053).

**Conclusion:**

RBA combined with AP nerve block provides adequate anesthesia for standing equine enucleation. US image quality and eyelid sensitivity are suitable block success predictors.

## 1 Introduction

Effective pain management through reliable local anesthesia is a fundamental requirement for surgical procedures in standing sedated horses [1]. Retrobulbar anesthesia (RBA) desensitizes the highly innervated eye and its adnexa by targeting sensory, motor, and autonomic nerve fibers within the retrobulbar space. This anatomically cone‐shaped region, bordered by the globe and extraocular muscles, contains the optic nerve (cranial nerve (CN) II), motor nerves (CN III, IV, VI), the ophthalmic and maxillary branches of the trigeminal nerve (CN V), the ciliary ganglion, vascular structures, retrobulbar fat, and the orbital fasciae. The auriculopalpebral (AP) branch of CN VII, responsible for eyelid closure, courses externally along the zygomatic arch and requires additional blockade [2].

Anatomically, RBA combined with an AP nerve block should provide sufficient anesthesia for enucleation in the standing, sedated horse. Nevertheless, published clinical protocols [2, 3, 4, 5, 6, 7, 8] frequently include additional periocular nerve or line blocks, and a recent survey among equine practitioners and ophthalmologists confirmed considerable variability in practice [9].

Reported complications of RBA relevant to enucleation include lack of anesthesia, intravascular injection, retrobulbar cellulitis, abscess formation, retrobulbar swelling, hypersensitivity reactions, and potentially life‐threatening events such as the oculocardiac reflex and intrameningeal injection [10].

Ultrasound (US)‐guided nerve blocks are increasingly utilized in both human and veterinary medicine. This technique enables real‐time visualization of relevant anatomical structures, allowing for accurate needle placement based on internal landmarks rather than variable external surface anatomy. Cadaveric studies in horses [11, 12, 13] and donkeys [14] have demonstrated the feasibility of US‐guided RBA and shown promising results regarding safety [12] and precision [11] compared to the blind technique. Notably, events such as ocular penetration [12] and masseter muscle injections [11] occurred in the blind groups, but not in the US‐guided groups. More recently, studies in live horses [15] and donkeys [16] have confirmed the feasibility of transpalpebral US‐guided dorsal RBA in clinical cases.

This study evaluated whether RBA combined with an AP nerve block provides sufficient anesthesia for standing equine enucleation. Moreover, it evaluated US‐guided needle positioning time, the effect of US guidance on block effectiveness, and the predictive value of image‐based scoring and sensory testing on surgical tolerance.

## 2 Material and Methods

### 2.1 Patients

The horses included in this study were part of the regular patient population of the equine clinic at Freie Universität Berlin between May 2021 and June 2024. Horses with end‐stage ocular disease necessitating enucleation were included. Cases of retrobulbar tumors, orbital abscesses, and panophthalmitis were excluded. Information was gathered regarding the animals' age, weight, sex, breed, and the clinical indications for enucleation.

### 2.2 Study Design

In a prospective clinical case series, the feasibility of performing enucleation using dorsal RBA combined with an AP nerve block and the impact of US assistance on RBA success were evaluated. Thirty horses received a standardized management (Section 2.2.1), medication (Section 2.2.2), sedation (Section 2.2.3), anesthetic nerve blocks (Section 2.2.4), surgical protocol and postoperative pain scoring (Section 2.2.5), (Figure 1). In Group 1 (*n* = 15) the dorsal RBA was performed using transpalpebral US assistance, whereas in the subsequent control Group 2 (*n* = 15) the procedure was performed blindly (Figure 2). For practical reasons, the investigator (SL) and the surgeon could not be blinded to group allocation.

**FIGURE 1:**
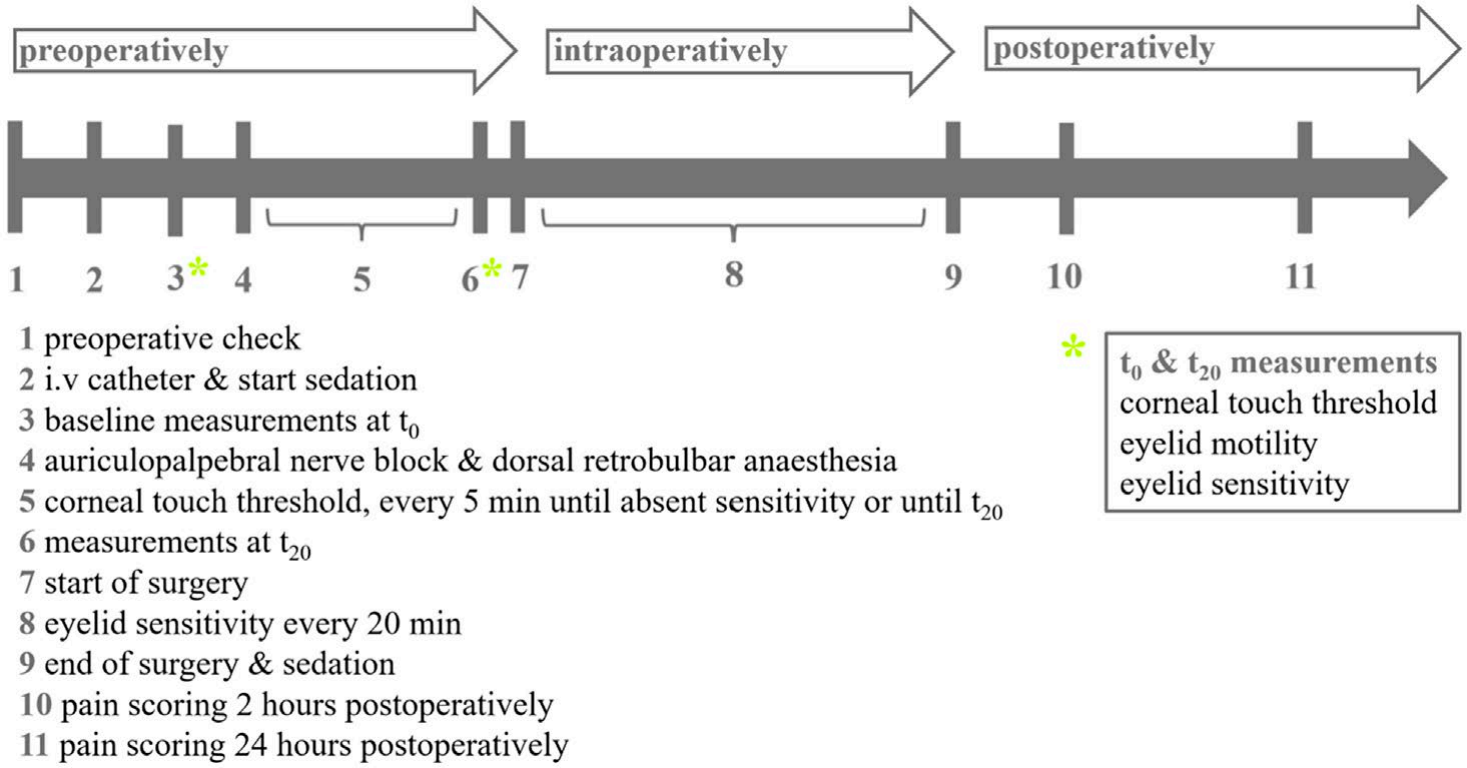
Study flow and measurement points in a prospective clinical trial of standing sedated enucleation in horses (*N* = 30). *t*_0_ is defined as the moment at which baseline values are obtained, preceding auriculopalpebral and dorsal retrobulbar anesthesia placement. *t*_20_ is defined as 20 min after block administration.

**FIGURE 2:**
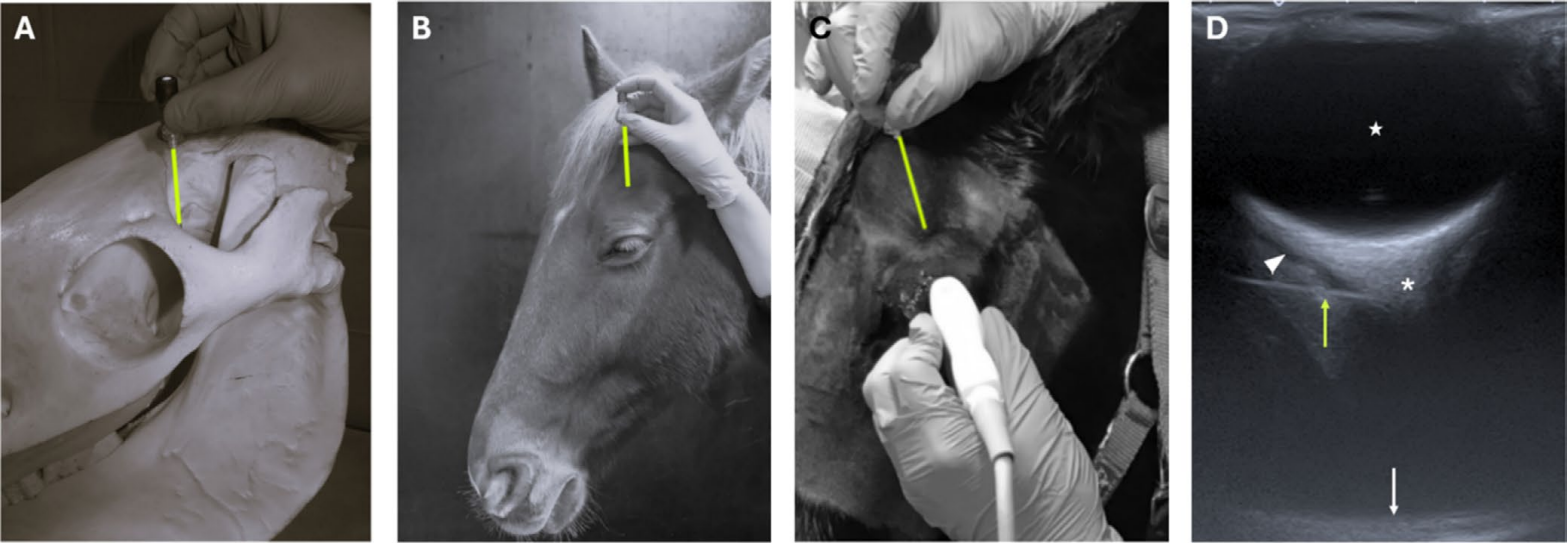
Photographs illustrating placement of a spinal needle (green line) for dorsal retrobulbar anesthesia (RBA) on (A) an equine skull, (B) an equine head, and (C) an equine head during ultrasound‐guided needle placement. (D) Ultrasound image during dorsal RBA in a horse using the ultrasound‐guided injection technique shown in (C), demonstrating excellent visualization according to the Morath et al. scoring system: Eyeball (star), retrobulbar muscle cone with intraconal space (arrowhead), optic nerve (asterisk), bony orbital shadow (white arrow), and needle (green arrow) visualized simultaneously.

Time is reported in minutes, with *t*_0_ defined as the moment at which baseline values are obtained, preceding AP and RBA placement. Subsequent points (*t*_5_, *t*_10_, etc.) denote 5, 10, etc. minutes after finishing block placement, respectively. Time used for RBA placement was measured separately and excluded from this count (Figure 1).

#### 2.2.1 Management

Horses were kept as in‐patients in clinic stalls. In addition to their routine diet, they received grain‐free mash containing paraffin oil pre‐ and postoperatively as colic prophylaxis, and hay nets were elevated postoperatively to reduce fluid accumulation in the wound cavity.

Horses not directly referred for enucleation underwent complete ophthalmic examination, including neuro‐ophthalmic reflex testing (menace, dazzle, palpebral and pupillary light reflexes), slit‐lamp biomicroscopy (SL‐17, Kowa Co), direct ophthalmoscopy (Heine BETA 200 LED, Heine Optotechnik), tonometry (Tonovet, Icare Finland Oy), fluorescein staining, and ultrasonography when indicated.

Preoperative assessment included a full physical examination and hematology (complete blood count, hematocrit, total protein). Tetanus vaccination status was verified by reviewing the vaccination record and, when uncertain, by titer testing (TetaCheck, Fassisi GmbH).

Postoperative monitoring followed an intensive care protocol, with vital parameters assessed every 6 h for 48 h.

#### 2.2.2 Medication

Flunixin‐meglumine was given i.v. at the start of surgery (1.1 mg/kg; Flunidol 50 mg/mL, CP‐Pharma) and orally postoperatively (1.1 mg/kg q12 h for 48 h; Flunidol 50 mg/mL, CP‐Pharma). Omeprazole (4 mg/kg; GastroGard, Boehringer Ingelheim) was administered orally once preoperatively and once postoperatively at a 24‐h interval. Antibiotics were not routinely administered and were given only at the surgeon's discretion.

#### 2.2.3 Sedation

Each patient received an intravenous catheter immediately prior to surgery. Detomidine hydrochloride (0.01 mg/kg i.v.; Cepesedan 10 mg/mL, CP‐Pharma) and butorphanol (0.02 mg/kg i.v.; Butorgesic 10 mg/mL, CP‐Pharma) were administered as an initial bolus in the stable. Upon positioning in the restraint stocks, a constant rate infusion (CRI) of the same drugs at the same concentrations per hour was started. If deeper sedation was required, the CRI rate was adjusted or an additional bolus of detomidine hydrochloride (0.005–0.01 mg/kg) was given. In the event of a defensive reaction, butorphanol (0.01 mg/kg) was administered.

Every 10 min, heart and respiratory rates were recorded, and sedation depth and quality were assessed using the scoring system described by Marly et al. [17]. The CRI rate was halved at the start of subcutaneous suturing and discontinued at the start of cutaneous suturing. Each patient received 5 L of a full electrolyte solution (Ringer‐Fresenius CFX DUO, Fresenius Kabi).

#### 2.2.4 Local Anesthetic Blocks

All patients received an AP nerve block and a dorsal RBA. These blocks were administered by the surgeon performing the enucleation, who was either a resident with a minimum of 18 months of specialty training or a diplomate of either the European College of Veterinary Ophthalmologists (ECVO) or the European College of Veterinary Surgeons (ECVS). All operators had moderate experience in performing US examinations, with at least 3 years of professional experience in the equine medicine field.

The following parameters were assessed prior to (*t*_0_) and 20 min after (*t*_20_) block administration: eyelid motility, eyelid sensitivity, and corneal sensitivity. Additionally, corneal sensitivity was assessed every 5 min between *t*_1_ and *t*_20_ until complete loss of corneal sensation, and eyelid sensitivity was measured at 20‐min intervals throughout the surgery.

##### 2.2.4.1 AP Nerve Block

For the motoric AP nerve block, 4 mL of 2% lidocaine hydrochloride (Bela‐Pharm) was administered at two injection sites using a 16 mm, 22G cannula (B. Braun, B. Braun Melsungen AG) following antisepsis with a 70% ethanol swab. The first injection was performed subfascially in the depression just anterior to the base of the ear (2 mL), and the second subcutaneously at the lowest curvature of the cranial zygomatic arch (2 mL) [18].

Success of the AP nerve block was defined as eyelid akinesia, assessed visually after tactile stimulation at *t*_10_ and/or *t*_20_. If the block was unsuccessful, it was repeated with the same injection technique at *t*_10_ and/or *t*_20_ as necessary, ensuring eyelid akinesia prior to surgery.

##### 2.2.4.2 RBA

The supraorbital fossa was aseptically prepared using a soap‐povidone‐iodine solution (1:3 ratio of Softaskin and VetSept, B. Braun Melsungen), followed by swabbing with 70% ethanol. A 70 mm, 22G cannula (Spine‐Ject, N. V.) was inserted under sterile conditions immediately posterior to the dorsal orbital rim and directed caudoventrally toward the contralateral upper molars [19, 20]. The aim was to position the needle tip within the intraconal space using either US guidance (Group 1) or anatomical landmarks only (Group 2). Once the needle was positioned, the stylet was removed, and a syringe containing the anesthetic was attached. Aspiration was performed prior to injection to confirm the absence of blood (i.e., to rule out intravascular needle placement), followed by administration of 12 mL of mepivacaine hydrochloride (Intra‐Epicaine, Dechra Regulatory B.V.) (Figure 2).

Success of RBA was defined as surgical tolerance at *t*_20_.

Surgical tolerance was considered to be present when, at the discretion of the surgeon, the horse tolerated the surgical procedure without exhibiting signs of discomfort, pain, or adverse reactions in response to the surgical stimulus.

If the RBA was unsuccessful at *t*_20_, anesthesia was established using subcutaneous infiltration of the eyelids. If the RBA became insufficient later during surgery, supplementary local anesthesia was administered at the surgeon's discretion as either a deep orbital splash block or infiltration anesthesia of the orbital tissues.

###### 2.2.4.2.1 US Assistance in Group 1

In Group 1 (*n* = 15), the dorsal RBA was applied with US assistance (Toshiba Aplio 500 ultrasonography unit with a 10 MHz linear US transducer, Toshiba Medical Systems Corporation) for needle positioning. The probe was positioned on the closed upper eyelid in a vertical orientation (Figure 2).

Imaging depth was set at 8.5 cm with the focus targeting the posterior globe at 5 cm.

Time required for needle positioning was defined as the interval from taking hold of the US probe to withdrawing the needle.

US image quality and the distribution pattern of the local anesthetic were assessed during acquisition by one investigator (SL) not involved in the surgical procedure, using the scoring system described by Morath et al. [13], classifying image quality as “excellent,” “good,” or “poor” and distribution as “excellent,” “satisfactory,” or “unsatisfactory”.

###### 2.2.4.2.2 Eyelid Sensitivity

Eyelid sensitivity was assessed at four predefined periocular sites, corresponding to the innervation territories of the supraorbital, lacrimal, zygomatic, and infratrochlear nerves. These sites were located approximately 20 mm from the midpoint of the upper and lower eyelid margins and the lateral and medial canthi, respectively (Figure 3). Sensitivity was evaluated by gently grasping a small skin fold with a mosquito clamp closed to the first notch of its locking mechanism. If no response was observed during the first test, the eyelid area was considered anesthetized. If the patient responded to the stimulus, the test was repeated three times, with a minimum of two positive responses indicating intact eyelid sensitivity. Eyelid sensitivity was assessed every 20 min throughout the surgery.

**FIGURE 3:**
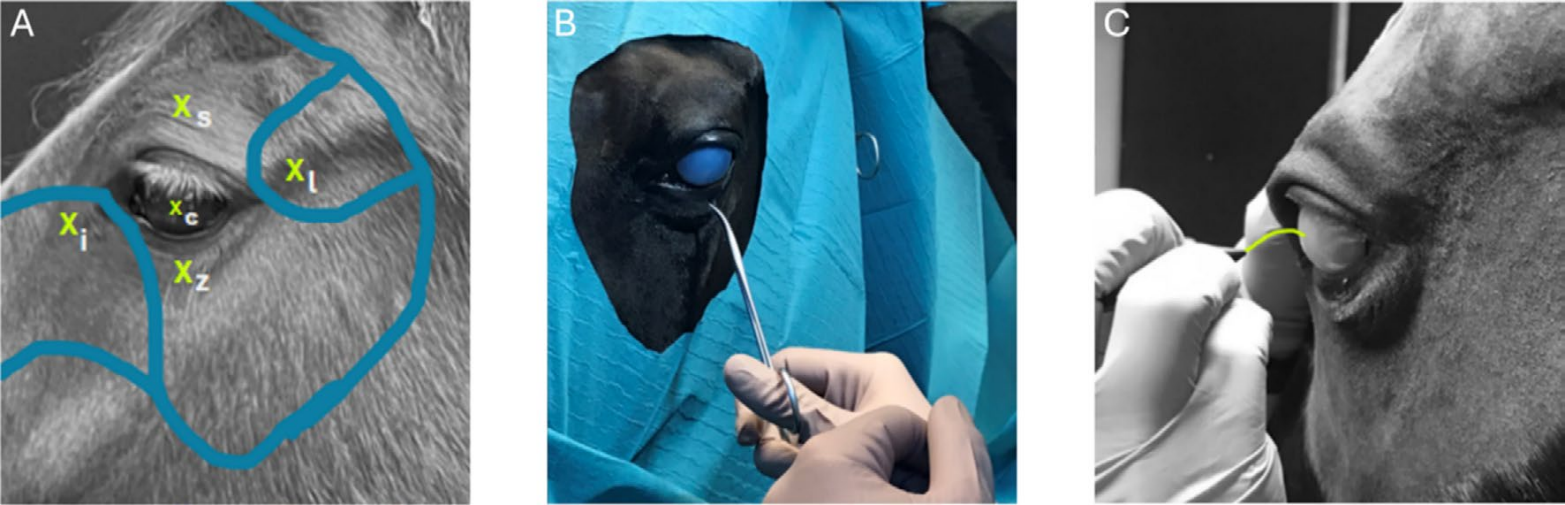
Photographs of an equine head depicting (A) periocular sensory innervation territories (blue lines) and designated measurement points (X) for eyelid sensitivity testing of the supraorbital (s), lacrimal (l), zygomatic (z), and infratrochlear (i) nerves, as well as the measurement point for the corneal touch threshold (c); (B) measurement of eyelid sensitivity; and (C) measurement of corneal touch threshold (green line indicates the nylon filament).

###### 2.2.4.2.3 Corneal Sensitivity

Corneal sensitivity was assessed using the corneal touch threshold (CTT) method [21] with a Cochet‐Bonnet aesthesiometer (nylon monofilament, 12/100 mm; Luneau, SAS). The filament was extended to 60 mm and applied perpendicular to the central cornea (Figure 3), causing a slight bend. Filament length was progressively reduced in 10 mm increments until reaching 40 mm, then in 5 mm increments until a blink reflex or defensive movement occurred in at least 2 of 3 stimuli. Filament length at this time was recorded in millimeters, with shorter measurements indicating reduced sensitivity.

Corneal sensitivity was assessed before local blocks (*t*_0_), immediately after block administration (*t*_1_), and every 5 min after block administration (*t*_5_, *t*_10_, *t*_15_) until complete corneal anesthesia was achieved and at *t*_20_.

#### 2.2.5 Surgery and Postoperative Painscoring

Surgery started at *t*_20_ and was performed in restraint stocks with the head supported on Styrofoam blocks. During the 20‐min interval between block administration and the start of surgery, the surgical site was aseptically prepared.

A transpalpebral or transconjunctival enucleation approach was chosen based on the indication for surgery or the surgeon's preference. Surgery time was defined as the interval from the initiation of palpebral suturing (for the transpalpebral approach) or lateral canthotomy (for the transconjunctival approach) to the completion of the stent suture.

Postoperatively, pain was assessed at 2 and 24 h using the EQUUS‐FAP score (Equine Utrecht University Scale for Facial Assessment of Pain) developed by van Loon et al. [22].

(Supporting Information S1). The criterion “eyelids” was not scored, resulting in a maximum possible score of 16 in this study instead of 18, as in the original score.

### 2.3 Data Analysis

Data were recorded in Microsoft Excel (version 2022; Microsoft Corp.). Statistical analyses were performed using IBM SPSS Statistics (version 29.0; IBM Corp.).

Descriptive statistics were used to summarize patient characteristics.

The distribution of continuous variables, including vital parameters, sedation and surgery time, time required for US‐guided needle placement, corneal sensitivity and postoperative pain scoring was assessed for normal distribution using the Shapiro–Wilk test. Depending on data distribution, group comparisons were performed using either a Student's *t*‐test (for normally distributed variables: sedation and surgery time) or a Mann–Whitney *U*‐test (for non‐normally distributed variables: corneal sensitivity and postoperative pain scoring values between groups or RBA block success). Corneal sensitivity values at different time points within a group were compared using the Wilcoxon test. Interquartile ranges (IQR) are provided wherever necessary.

Categorical variables, including US image quality, distribution pattern of the local anesthetic, and postoperative pain scores between groups were analyzed using Chi‐square tests. Surgery method, RBA success rates, and eyelid sensitivity were evaluated using Fisher's exact tests.

Additionally, a linear by linear association test (based on Mantel Haenszel test) was applied to assess the presence of a linear trend between US image quality and block success and between local anesthetic distribution pattern and block success.

The association between eyelid sensitivity and RBA block success at *t*_20_ was evaluated using binary logistic regression, reporting regression coefficients (b), odds ratios (OR), and 95% confidence intervals (CI).

For all analyses, a *p*‐value < 0.05 was considered statistically significant.

OR and 95% CI were reported where appropriate.

## 3 Results

### 3.1 Study Design and Patients

The two groups were comparable in terms of mean age, weight, sex, breed, and indications for enucleation. Group 1 had a mean age of 19.4 years and a mean body weight of 487.5 kg, comprising 7 mares and 8 geldings. Group 2 had a mean age of 19.5 years and a mean body weight of 464.0 kg, comprising 6 mares and 9 geldings.

Indications for enucleation in Group 1 included equine recurrent uveitis (5), uveitis (3), immune‐mediated keratitis (3), corneal ulcer (1), conjunctival squamous cell carcinoma (1), glaucoma (1), and endophthalmitis (1). In Group 2, indications were equine recurrent uveitis (5), uveitis (3), immune‐mediated keratitis (1), corneal ulcer (4), glaucoma (1), and globe perforation (1).

Seven surgeons participated in Group 1 and five in Group 2, with two surgeons contributing to both groups.

### 3.2 Evaluation of Local Anesthetic Blocks

#### 3.2.1 AP Nerve Block and Eyelid Motility

Prior to AP nerve block administration, eyelid motility was present in all patients. At *t*_10_, eyelid akinesia was observed in 73.3% (22/30) of cases in both groups. In the eight initially unsuccessful cases, the block was repeated using the same injection technique. In 7/8 cases, eyelid akinesia was achieved after a single reapplication; one case needed a second reapplication. Eyelid akinesia was successfully established in all patients prior to surgery.

#### 3.2.2 RBA

##### 3.2.2.1 Success of RBA

Initial RBA was successful, with success defined as surgical tolerance at *t*_20_, in 63.3% of horses (19/30). The success rate was 73.3% in Group 1 (11/15; 95% CI, 48.1–89.1) and 53.3% in Group 2 (8/15; 95% CI, 30.1–75.2). This difference did not reach statistical significance (*p* = 0.179).

Among the 19 initially successful cases, 84.2% (16/19) remained effective until the end of surgery. Only 3/19 horses required an additional deep intraorbital injection of local anesthetic during transection of the retrobulbar tissues between *t*_60_ and *t*_80_ (Table 1, Figure 4).

**TABLE 1:**
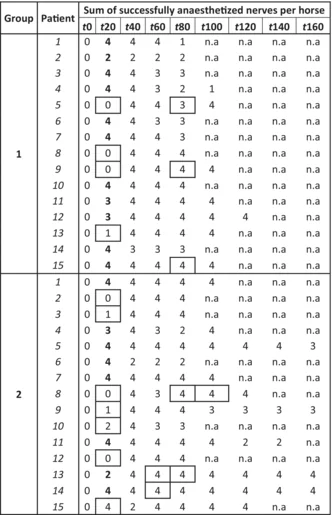
Number of periocular innervation territories successfully anesthetized before (*t*_0_) and at 20–160 min (*t*_20_–*t*_160_) following dorsal retrobulbar anesthesia (RBA) combined with an auriculopalpebral nerve block.

*Note:* Results are shown as absolute numbers for Group 1 (ultrasound‐guided dorsal RBA) and Group 2 (blind dorsal RBA). Data collection was discontinued after surgery (n.a., not applicable). Bold values at *t*_20_ denote surgical tolerance. Boxed values indicate cases requiring additional local anesthetic to achieve surgical tolerance.

**FIGURE 4:**
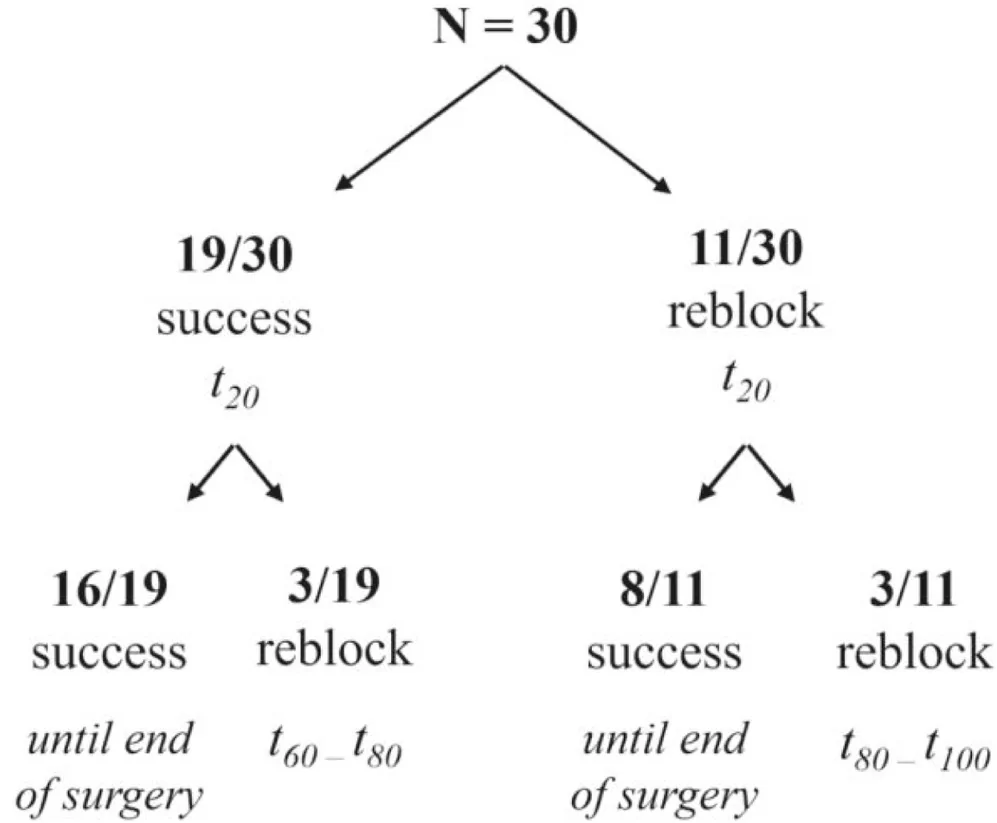
Flowchart illustrating success rates of dorsal retrobulbar anesthesia (RBA) in horses enrolled in a prospective clinical trial (*N* = 30). Time points (*t*) indicate minutes after RBA placement (e.g., *t*_20_ = 20 min).

In 11/30 cases that were initially unsuccessful, local anesthesia was achieved through additional subcutaneous eyelid nerve blocks administered at the surgeon's discretion before the start of surgery (*t*_20_–*t*_30_). Three of these cases required an additional injection of local anesthetic deep into the orbit during transection of retrobulbar tissue between *t*_80_ and *t*_100_ (Table 1, Figure 4).

##### 3.2.2.2 US Assistance in Group 1

The mean time required for US‐assisted RBA was 5.2 min (±1.7 min; range: 2.3–8.3 min).

US image quality was rated as “excellent” in 3/15 cases, “good” in 7/15, and “poor” in 5/15 cases, with corresponding success rates of 100% (3/3), 85.7% (6/7), and 40% (2/5), respectively. Although not statistically significant, the RBA block success rates increased with improving image quality (*p* = 0.053).

The distribution pattern of the local anesthetic was graded as “excellent” in 3/15 cases, “satisfactory” in 10/15, and “unsatisfactory” in 2/15 cases, with success rates of 66.7% (2/3), 80% (8/10), and 50% (1/2), respectively. No statistically significant association with RBA success was observed (*p* = 0.793).

##### 3.2.2.3 Corneal Sensitivity

At *t*_0_, the median CTT was 25.0 mm (IQR: 7.5–35.0 mm) in Group 1 and 15.0 mm (IQR: 5.0–25.0 mm) in Group 2 (Figure 5). At *t*_1_, CTT values dropped directly from baseline to 0.0 mm in 80% (12/15) of patients in both groups. At *t*_15_, no recordable corneal sensitivity was observed in any of the horses without further drug administration. Individual CTT progressions are shown in Figure 5.

**FIGURE 5:**
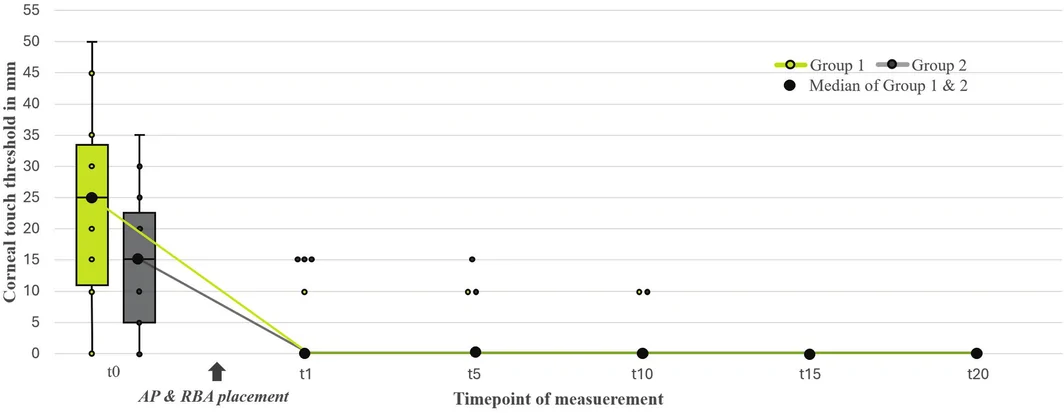
Longitudinal plot showing the median and individual outlier values of the corneal touch threshold after dorsal retrobulbar anesthesia (RBA) and auriculopalpebral (AP) nerve block in horses undergoing standing enucleation. At *t*_0_, baseline measurements are depicted as boxplots. Time points (*t*) represent minutes after block placement, with *t*_0_ as the baseline and *t*_1_, *t*_5_, *t*_10_, and *t*_20_ corresponding to 1, 5, 10, and 20 min, respectively. Group 1 (*n* = 15) received the RBA with transpalpebral ultrasound guidance. Group 2 (*n* = 15) underwent a blind procedure.

CTT values were significantly lower at *t*_1_ and *t*_20_ compared to baseline in both groups (*p* < 0.01 for both Wilcoxon tests). There was no statistically significant association between CTT measurements and the success of RBA.

Due to patient non‐compliance, the following measurement points are missing in Group 1:

*t*_0_ from one patient and *t*_1_–*t*_15_ from two patients.

##### 3.2.2.4 Eyelid Sensitivity

At *t*_0_, eyelid sensitivity was present in all cases in both groups (Table 1).

At *t*_20_, in Group 1, 3/1/1/2/8 out of 15 horses had 0/1/2/3/4 anesthetized innervation territories (Table 2), respectively. In Group 2, 3/2/2/1/7 out of 15 horses had 0/1/2/3/4 anesthetized innervation territories, respectively. There was a significant association between the number of successfully anesthetized innervation territories per horse and the success of RBA (*p* < 0.001; Table 2) at *t*_20_. With each additionally anesthetized territory, the odds of achieving a successful RBA increased by a factor of 6.1 per nerve (*b* = 1.8, *p* = 0.003; OR = 6.1; 95% CI: 1.89–19.99).

**TABLE 2:** Association between the number of anesthetized periocular innervation territories (0–4) in standing sedated horses (*N* = 30) after the administration of a dorsal retrobulbar block and the success of retrobulbar anesthesia (RBA), defined as surgical tolerance 20 min after block administration (*t*_20_; *p* < 0.001) and stratified by groups.

Anesthetized innervation territories at *t*_20_	Total animals *N* = 30	RBA success (*t*_20_)							
		Yes *N* = 19				No *N* = 11			
*n*	*n*	*n* (G1)	*n* (G2)	*n*	%	*n* (G1)	*n* (G2)	*n*	%
4	15	8	6	14	93%	0	1	1	7%
3	3	2	1	3	100%	0	0	0	0%
2	3	1	1	2	67%	0	1	1	33%
1	3	0	0	0	0%	1	2	3	100%
0	6	0	0	0	0%	3	3	6	100%

Abbreviations: G1, Group 1 (*n* = 15); G2, Group 2 (*n* = 15).

Horses with unsuccessful RBA immediately received an additional application of local anesthetic, after which surgical tolerance was achieved. By *t*_40_, all patients tolerated surgery, having at least 2/4 innervation territories anesthetized.

Between *t*_60_ and *t*_100_, six horses required an additional injection of local anesthetic deep into the orbit during transection of retrobulbar tissue. Notably, eyelid anesthesia was still present in these patients, with 4/4 nerves anesthetized in 5/6 cases and 3/4 in 1/6 cases.

### 3.3 Sedation, Surgery, and Postoperative Pain Scoring

The median heart rate was 32 beats per minute in both groups (IQR: 28–36 in Group 1; 32–36 in Group 2). The median respiratory rate was 12 breaths per minute in both groups (IQR: 12–16 in both groups).

The mean sedation time was significantly longer in Group 2 (163.3 ± 26.8 min) than in Group 1 (117.0 ± 18.7 min), with a mean difference of 46.3 min (95% CI: 29.0–63.6 min; *p* < 0.001).

In Group 1, the transconjunctival technique was employed in 8/15 procedures and the transpalpebral technique in 7/15. The mean time for surgery was 68.5 ± 17.2 min.

In Group 2, the transconjunctival technique was applied in 1/15 surgeries and the transpalpebral technique was utilized in 14/15 surgeries. The mean time for surgery was 97.3 ± 28.0 min.

In Group 2, the transpalpebral technique was used significantly more frequently (*p* = 0.014, OR 15.9, 95% CI 1.7–166.7), and the mean duration of surgery was also significantly longer compared to Group 1 (mean difference: 28.8 min, 95% CI: 11.4–46.2 min; *p* = 0.002).

The EQUUS‐FAP score revealed no significant differences between the two groups (Figure 6). Two hours postoperatively, the scores ranged from 0–6/16 in Group 1 (median 1) and from 0–5/16 in Group 2 (median 2). Twenty‐four hours postoperatively, the scores ranged from 0–5/16 in Group 1 (median 1) and from 0–1/16 in Group 2 (median 0). Whether the initial RBA block was successful or not had no effect on the score.

**FIGURE 6:**
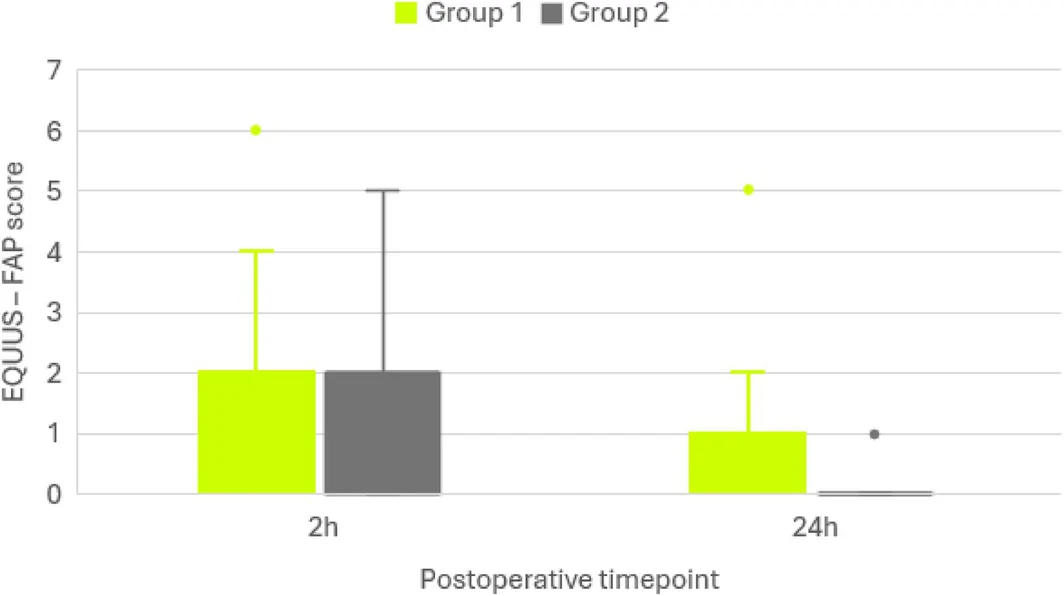
Boxplot illustrating postoperative pain scores at 2 and 24 h following enucleation performed under standing sedation in a prospective clinical study. Pain was assessed using the EQUUS‐FAP (Equine Utrecht University Scale for Facial Assessment of Pain) as described by van Loon et al. Due to omission of one criterion, the maximum achievable score in this study was 16 points rather than 18. Group 1 (*n* = 15) received the RBA with transpalpebral ultrasound guidance. Group 2 (*n* = 15) underwent a blind procedure.

## 4 Discussion

### 4.1 RBA and AP Sufficient for Enucleation

To the authors' knowledge, this is the first study to demonstrate that combined RBA and AP nerve block can provide sufficient anesthesia and analgesia for enucleation in the standing sedated equine patient. Notably, 53.3% (16/30) of horses required only these initial blocks for the entire surgical procedure.

From an anatomical perspective, this combination is expected to offer adequate regional anesthesia, as all relevant nerves, with the exception of the AP nerve, exit the posterior aspect of the bony orbit [2, 20]. Nevertheless, several published protocols recommend the use of additional local nerve blocks for this procedure. Pollock [3] and Tóth [8] described approaches with RBA, AP, supraorbital, lacrimal, zygomatic, and infraorbital nerve blocks. In Gilger's *Equine ophthalmology*, Hartley and Grundon [2] further recommend topical corneal anesthesia. Auer [6] proposed a combination of the aforementioned nerve blocks, excluding the zygomatic nerve block and corneal anesthesia. Hewes [7] employed RBA and a supraorbital nerve block, supplemented by lower eyelid infiltration. Townsend [5] and Henriksen [4] used the same combination and additionally applied AP, topical corneal anesthesia, and upper eyelid infiltration.

A recent survey of equine and ophthalmic veterinarians demonstrated a wide variation in local anesthetic protocols for enucleation in standing, sedated patients. The 238 participants reported 65 unique nerve block combinations [9], which demonstrate the lack of a standardized approach.

### 4.2 RBA Success

Overall RBA success at *t*_20_ was 63.4% (19/30), with rates of 73.3% (11/15) in Group 1 and 53.3% (8/15) in Group 2 (*p* = 0.179). The definition of “success” as “surgical tolerance” at *t*_20_ represents a particularly stringent standard for block success, as it is not limited to isolated sensory testing but reflects the overall clinical adequacy of anesthesia during surgery.

As this assessment is subject to the surgeon's discretion, the success rates may have been influenced by personal bias.

The higher success rate observed in Group 1 may be explained by several factors.

First, US guidance may have improved the accuracy of needle placement and local anesthetic deposition, thereby increasing the likelihood of a successful RBA [11, 12].

Second, the distribution of surgical approaches differed between groups. In the transconjunctival approach, adequate eyelid anesthesia at the start of surgery is less critical than in the transpalpebral approach, where placement of the eyelid sutures represents an early and intense surgical stimulus [20]. The transconjunctival technique was performed in 8/15 horses in Group 1 compared with only 1/15 horses in Group 2. This imbalance in surgical approach may therefore have contributed to the higher success rate observed in Group 1. Consistent with these findings, previous studies have reported reliable corneal anesthesia following RBA, whereas eyelid anesthesia has been more variable [15, 21].

Collectively, these findings suggest that RBA combined with an AP nerve block provides sufficient anesthesia for transconjunctival enucleation. In contrast, the transpalpebral approach may require a longer interval between administration of the nerve block and eyelid suturing, or supplementary local infiltration of the eyelids, to achieve adequate surgical anesthesia.

Regardless of group allocation, 11/30 horses had not achieved a sufficient level of anesthesia for surgical tolerance at *t*_20_, likely because the local anesthetic did not reach the desired destination.

Following additional subcutaneous infiltration of local anesthetic into the eyelids at *t*_20_, 8/11 horses achieved adequate anesthesia for the remainder of the surgical procedure.

The remaining 3/11 unsuccessful cases and 3/19 initially successful cases required an additional top up of local anesthetic deep into the orbit 60–100 min after the initial RBA. Interestingly, horses that had shown initial success required reapplication of a local anesthetic between *t*_60_ and *t*_80_, whereas those that had already required an additional reapplication at *t*_20_ needed it later, between *t*_80_ and *t*_100_ (Table 1). These time frames correspond well with the reported elimination half‐life of mepivacaine (approximately 1.5–2 h [1]), supporting the plausibility of waning anesthetic effect during this period.

Another explanation for the required top up may be the heterogeneous anatomical course of the zygomatic, supraorbital, lacrimal, and infratrochlear nerves, which may not have been consistently reached by the local anesthetic [10].

### 4.3 US Assistance and Complications

Although it did not reach statistical significance, the success rate was higher with US‐guided RBA than with the blind technique, and improved US image quality also led to a higher success rate (*p* = 0.053). There was a 100% success rate with “excellent” US images compared to a 40% rate with “poor” images. This finding is supported by several equine cadaveric studies. Morath et al. [13] and Thieme et al. [12] evaluated the use of US guidance for the dorsal block, while Leigh et al. [11] investigated a peribulbar approach. Two of these studies [11, 12] compared US‐guided and blind injections and demonstrated that US assistance improved needle placement accuracy.

Although US guidance may increase the time required for needle positioning, we did not measure the time required for the blind technique and therefore cannot directly compare procedural times. Needle positioning is required for both techniques and was included in our US time measurements, so a simple addition of the time for US needle placement would not be adequate. In addition, US setup time varies between clinics. While integration of US into routine workflows may initially be challenging, efficiency is likely to improve with experience and streamlined workflows. Furthermore, if US guidance increases block success, it may reduce or eliminate repeat blocks, thereby offsetting the additional time required.

US‐guided nerve blocks are increasingly used in both human and veterinary medicine. Needle placement is based on real‐time visualization of internal structures rather than variable surface landmarks, enabling precise positioning and adjustment during injection for accurate nerve blockade [1]. Hermans et al. [23] demonstrated a training effect in equine cadavers, observing a trend toward significantly improved needle positioning following US‐guided training. The lack of prior US RBA training among the participating surgeons in our study, combined with the small sample size, may have contributed to our non‐significant findings.

Complications of RBA include hypersensitivity reactions, insufficient akinesia, compression of the surgical field, exophthalmos, keratitis, chemosis, globe perforation, intravascular injection, retrobulbar hemorrhage, cellulitis, abscess formation, optic nerve puncture, and life‐threatening events such as the oculocardiac reflex or intrameningeal injection [9, 10]. Some of these risks may be reduced with US‐guided needle placement, particularly among less experienced clinicians: either by avoiding unintended structures or by enabling more precise local anesthetic deposition, potentially reducing the required anesthetic volume [11, 12, 15].

### 4.4 Volume

The volume used for dorsal RBA in a recent survey [9] ranged from 2–30 mL (*n* = 192). This wide variation, together with the additional use of local nerve blocks of varying volumes, suggests that the administered volume may influence whether supplementary blocks are required. Consequently, both the surgical plan and the choice of blocks should be considered when determining the volume of local anesthetic. The volume that can be safely applied depends on the type of surgery. In procedures other than enucleation, a rise in intraocular pressure caused by RBA may pose a risk for compromised globes [15].

More recently, Tooley et al. [15] conducted a clinical study evaluating a low‐dose (5 mL) dorsal RBA using US guidance for needle placement. They demonstrated that administering a low volume at a precise location can achieve successful outcomes. Similar to our findings, they reported a significant decrease in corneal sensitivity one minute after RBA administration; however, satisfactory eyelid desensitization was not achieved.

Yang et al. [21] used 10 mL of lidocaine and likewise observed a significant decrease in corneal sensitivity, while eyelid desensitization was only partially successful. These results are consistent with our observations: even in horses with successful RBAs, the number of anesthetized innervation territories per horse varied between two and four.

The 12 mL volume used in our study appeared adequate for enucleation, where sufficient eyelid anesthesia is also required. However, for surgeries involving only the cornea or other globe‐sparing procedures, using a lower volume of local anesthetic may help reduce risks—particularly in cases with elevated intraocular pressure.

### 4.5 Evaluation of Predictors for RBA Success

#### 4.5.1 Corneal Sensitivity

As all horses exhibited absent corneal sensitivity at *t*_20_, CTT does not appear to be a useful predictor of overall block success in our study population. CTT success evaluation may have been confounded by the simultaneous application of an AP nerve block, as it was assessed before and after motor blockade. This limits comparability and reliability, since complete palpebral closure could no longer be expected following AP block administration. In 26/30 horses, CTT was absent immediately after block placement. In the remaining 4/30 cases, where corneal sensitivity was still partially present, avoidance responses such as head withdrawal were observed. This suggests that, despite motor blockade, CTT assessment retained diagnostic value.

CTT may be more appropriately interpreted as an indicator of the onset and extent of corneal anesthesia. This remains clinically relevant for procedures involving the cornea, including anterior chamber paracentesis, intrastromal injection, and corneal surgery, where reliable corneal desensitization is required.

#### 4.5.2 Eyelid Sensitivity

The evaluation of eyelid sensitivity was less affected by the AP block, as the typical response to cutaneous stimulation is not a blink reflex but head withdrawal. In other studies assessing the success of RBA in horses, von Frey filaments have been used to objectively measure eyelid sensitivity [15, 21], while additional methods of quantitative sensory testing have also been described [24]. In our study we opted for a mosquito clamp, as this method is easily integrated into the surgical workflow and may therefore facilitate its applicability and routine use in clinical practice.

#### 4.5.3 Mydriasis

In our study, mydriasis was not a reliable predictor of RBA success, as horses had end‐stage ocular disease often associated with impaired or fixed pupillary dilation (e.g., glaucoma, posterior synechiae, or atropine treatment). In contrast, previous studies in healthy horses identified mydriasis as a reliable indicator of successful RBA and oculomotor nerve blockade.

Adequate pupil dilation is necessary for posterior segment procedures such as cataract surgery and vitrectomy [15, 21], expanding the indications of RBA.

### 4.6 Sedation, Surgery, and Postoperative Pain‐Scoring

#### 4.6.1 Sedation

To maintain stable sedation, we followed the scoring system described by Marly et al. [17], which proved effective. Although butorphanol in sedated horses is known to cause side effects such as forward movement, increased locomotor activity, head shaking, and muzzle twitching [17], no adverse reactions affecting surgery were observed. The combination of an NSAID, an opioid, and an α_2_‐agonist likely influenced both pain perception and the expression of pain responses, potentially affecting sensory testing. Yang et al. [21] reported that detomidine at a similar dosage did not alter corneal sensitivity; however, the additional administration of NSAIDs and opioids in our study may have modulated pain perception. Nevertheless, clear responses to sensitivity testing were observed, retaining diagnostic value. As these reactions were comparable between the two groups, this supports the reliability of the clinical sedation protocol used.

#### 4.6.2 Surgery

The imbalance in surgical approaches between the two groups may have influenced the reported success rates due to differences in surgical stimulus (see Section 4.1). Additionally, the longer sedation and surgical times in Group 2 likely reflect the higher proportion of transpalpebral enucleations, as this technique generally requires more time than the transconjunctival approach.

#### 4.6.3 Pain Scoring

Overall, pain scores were low in both groups, supporting the conclusion that our protocol for enucleation, including medication, sedation, and local anesthesia, was well tolerated. The absence of significant differences in pain scores between groups is expected, as adequate surgical tolerance had been established in all animals, either directly at *t*_20_ or following additional interventions. Since the EQUUS‐FAP [22] score includes a criterion evaluating both eyes, this criterion was not scored, resulting in a maximum possible score of 16 points in this study, as opposed to 18 points in the original scoring system. The implications for comparability with studies using the original EQUUS‐FAP scale should be considered. When comparing our results with future studies using the original score, this modification of the scale should be taken into account, as it results in slightly higher relative pain scores (e.g., 5/16 vs. 5/18, corresponding to a 12.5% higher pain proportion in our study).

### 4.7 Anatomical Considerations and Clinical Implications

In our study, corneal sensitivity was reliably absent following the dorsal RBA injection of 12 mL of mepivacaine, regardless of block success—attributable to effective blockade of the long and short ciliary nerves within the intraconal space.

In contrast, lid sensitivity showed variable outcomes, as it depends on the extent of anesthesia of the zygomatic, supraorbital, lacrimal, and infratrochlear nerves, which potentially have more variable anatomical courses and may not be uniformly reached. Surgical tolerance occasionally existed despite incomplete anesthesia of all four nerves, likely due to sufficient block of key sensory branches or individual variability in nociceptive thresholds. Conversely, the presence of intolerance despite a complete nerve blockade, as observed in one patient in Group 2 (Table 1), may indicate incomplete perineural spread, anatomical variations, or central sensitization mechanisms.

Using alternative orbital injection techniques than dorsal RBA could result in different patterns of local anesthetic distribution, thereby potentially improving anesthesia. Extraconal peribulbar approaches have been investigated in cadaveric studies with [11] and without [25] US assistance. A Sub‐Tenon injection has been described in a cadaveric study [26] and used during cataract surgery and vitrectomy with promising results [27]; however, whether it provides sufficient anesthesia for the surgical stimulus of enucleation remains unclear. Further clinical in vivo studies are needed to determine whether these techniques offer more reliable surgical anesthesia.

### 4.8 Limitations

The consecutive and non‐blinded study design may have influenced the observed outcomes. However, this sequence was chosen for animal welfare reasons, as the feasibility of using RBA and AP as the sole local anesthetic techniques was first established under optimal conditions using US guidance. Pre‐existing ocular diseases as well as pre‐ and perioperative medication may additionally have affected the outcomes. Responses to CTT testing were potentially obscured by the use of an AP nerve block. Potential observer bias may have influenced the evaluation of US image quality and anesthetic distribution, as all assessments were performed by a single investigator (SL) without external validation. Surgical tolerance was a subjective parameter evaluated by the surgeon, likely introducing observer‐related bias that may have influenced the reported success rates. The small sample size may limit the statistical power and generalisability of the findings, warranting validation in a larger cohort.

## 5 Conclusion

RBA, in combination with an AP nerve block, sedation, and a non‐steroidal anti‐inflammatory drug, provides sufficient anesthesia and analgesia for enucleation in the standing, sedated equine patient, thereby eliminating the need for general anesthesia. The use of US guidance increases procedure time only marginally. Lid sensitivity and US image quality are suitable predictors of surgical tolerance and can be readily integrated into the surgical workflow. Future clinical studies in a larger cohort would be valuable to confirm these findings, with particular consideration of alternative injection techniques and the potential training effect of US assistance. Furthermore, the influence of different volumes or local anesthetic agents should be evaluated.

## Author Contributions

**Simona Lieberth:** conceptualization, methodology, data curation, investigation, formal analysis, funding acquisition, visualization, project administration, writing – original draft, validation. **Katharina Thieme:** conceptualization, investigation, validation, visualization, writing – review and editing, supervision. **Christian Dancker:** writing – review and editing, conceptualization, methodology. **Roswitha Merle:** formal analysis, methodology, validation, visualization, writing – review and editing. **Johanna Corinna Eule:** conceptualization, investigation, validation, visualization, supervision, resources, funding acquisition, writing – review and editing.

## Funding

The authors have nothing to report.

## Disclosure

*Artificial Intelligence Statement*: The authors have not used AI to generate any part of the manuscript.

## Ethics Statement

The study was approved by the local research ethics committee “Landesamt für Gesundheit und Soziales Berlin” (StN 043/23).

## Consent

Written owner consent was obtained for research and publication.

## Conflicts of Interest

The authors declare no conflicts of interest.

## Supporting information


**Data S1:** Postoperative pain scoring system, applied at 2 and 24 h following enucleation performed under standing sedation in a prospective clinical study (EQUUS‐FAP Equine Utrecht University Scale for Facial Assessment of Pain as described by van Loon et al.). Due to omission of one criterion (*italics*), the maximum achievable score in this study was 16 points rather than 18.

## Data Availability

The data that support the findings of this study are openly available in figshare at https://figshare.com/s/4323024b3aa52dd79749.

## References

[1] G. E. Rioja , “Local Anesthetics,” in Veterinary Anesthesia and Analgesia: The Fifth Edition of Lumb and Jones, Fifth Edition ed., ed. K. A. Grimm LAL , W. J. Tranquilli , S. A. Greene , and S. A. Robertson (John Wiley & Sons, Inc, 2015), 332–354.

[2] C. Hartley and R. A. Grundon , “Diseases and Surgery of the Globe and Orbit,” in Equine Ophthalmology, Fourth Edition ed., ed. B. C. Gilger (Wiley Blackwell, 2022), 133–186.

[3] P. J. Pollock , T. Russell , T. K. Hughes , M. R. Archer , and J. D. Perkins , “Transpalpebral Eye Enucleation in 40 Standing Horses,” Veterinary Surgery 37, no. 3 (2008): 306–309. 10.1111/j.1532-950X.2008.00382.x 18394080

[4] M. de Linde Henriksen and D. E. Brooks , “Standing Ophthalmic Surgeries in Horses,” Veterinary Clinics of North America. Equine Practice 30, no. 1 (2014): 91–110. 10.1016/j.cveq.2013.11.012 24680208

[5] W. M. Townsend , How to Perform a Standing Enucleation. Proceedings of the 59th Annual Convention of the American Association of Equine Practitioners (AAEP, 2013).

[6] J. A. Auer , J. A. Stick , J. M. Kümmerle , et al., Equine surgery, Fifth Edition ed. (Elsevier, 2019).

[7] C. A. Hewes , G. C. Keoughan , and S. Gutierrez‐Nibeyro , “Standing Enucleation in the Horse: A Report of 5 Cases,” Canadian Veterinary Journal 48, no. 5 (2007): 512–514. PMC1852602 17542371

[8] J. Tóth , J. Hollerrieder , C. van der Geer , and S. Huthmann , “Transpalpebrale Bulbusentfernung am Stehenden Pferd,” Tierärztliche Praxis. Ausgabe G, Grosstiere/Nutztiere 36, no. 2 (2008): 119–123.

[9] S. Lieberth , K. Thieme , C. Dancker , R. Merle , and J. C. Eule , “Global Survey on the Utilisation and Experiences With Different Retrobulbar Anaesthesia Techniques in Horses,” Equine Veterinary Journal 58 (2025): 1091–1102. 10.1111/evj.70082 PMC13244178 40847632

[10] R. A. Mc Kinney , “The Retrobulbar Block: A Review of Techniques Used and Reported Complications,” Equine Veterinary Education 33, no. 6 (2021): 332–336.

[11] H. Leigh , M. Gozalo‐Marcilla , V. Esteve , et al., “Description of a Novel Ultrasound Guided Peribulbar Block in Horses: A Cadaveric Study,” Journal of Veterinary Science 22, no. 2 (2021): e22, https://www.ncbi.nlm.nih.gov/pubmed/33774938. 10.4142/jvs.2021.22.e22 PMC8007447 33774938

[12] K. Thieme , L. Mesquita , S. Lieberth , C. Dancker , M. G. Doherr , and J. C. Eule , “Safety and Accuracy of Blind vs. Ultrasound‐Guided Dorsal Retrobulbar Nerve Blocks in Horses‐A Cadaveric Study,” Veterinary Ophthalmology 26, no. 2 (2023): 135–144. 10.1111/vop.13053 36536509

[13] U. Morath , C. Luyet , C. Spadavecchia , M. H. Stoffel , and G. M. Hatch , “Ultrasound‐Guided Retrobulbar Nerve Block in Horses: A Cadaveric Study,” Veterinary Anaesthesia and Analgesia 40, no. 2 (2013): 205–211. 10.1111/j.1467-2995.2012.00780.x 23057999

[14] U. Hagag and A. El Nahas , “Ultrasound‐Guided Retrobulbar Nerve Block in Donkey Cadavers,” Veterinary Anaesthesia and Analgesia 49, no. 2 (2022): 197–202. 10.1016/j.vaa.2022.01.002 35148935

[15] Z. C. Tooley , J. S. Eaton , S. M. Grant‐Counard , S. M. Ilkenhans , S. J. Hetzel , and K. J. Bartholomew , “Comparative Efficacy of Low‐Volume Retrobulbar Anesthesia Using Three Commercial Local Anesthetics in Adult Horses,” Journal of the American Veterinary Medical Association 1 (2024): 1–11. 10.2460/javma.24.02.0121 38701834

[16] S. Gadallah , A. Sharshar , E. Tageldeen , and A. Hammad , “Ultrasound‐Guided Auriculopalpebral and Retrobulbar Nerve Blocks in Donkeys,” Journal of Current Veterinary Research 6, no. 2 (2024): 18–29.

[17] C. Marly , R. Bettschart‐Wolfensberger , P. Nussbaumer , S. Moine , and S. K. Ringer , “Evaluation of a Romifidine Constant Rate Infusion Protocol With or Without Butorphanol for Dentistry and Ophthalmologic Procedures in Standing Horses,” Veterinary Anaesthesia and Analgesia 41, no. 5 (2014): 491–497. 10.1111/vaa.12174 24835127

[18] A. E. Dwyer and M. de Linde Henriksen , “Equine ocular examination and treatment techniques,” in Equine Ophthalmology, Fourth edition ed., ed. B. C. Gilger (Wiley Blackwell, 2022), 1–89.

[19] R. Stoppini and B. C. Gilger , “Equine ocular examination basic techniques,” in Equine Ophthalmology, ed. B. C. Gilger (Wiley Blackwell, 2016), 1–39.

[20] K. N. Gelatt , J. P. Gelatt , and C. Plummer , Veterinary Ophthalmic Surgery‐E‐Book: Veterinary Ophthalmic Surgery‐E‐Book (Elsevier Health Sciences, 2021).

[21] V. Y. Yang , J. S. Eaton , K. Harmelink , et al., “Retrobulbar Lidocaine Injection via the Supraorbital Fossa Is Safe in Adult Horses but Produces Regionally Variable Periocular Anaesthesia,” Equine Veterinary Journal 54 (2021): 807–819. 10.1111/evj.13496 34396584

[22] J. P. van Loon and M. C. Van Dierendonck , “Monitoring Equine Head‐Related Pain With the Equine Utrecht University Scale for Facial Assessment of Pain (EQUUS‐FAP),” Veterinary Journal (London, England: 1997) 220 (2017): 88–90. 10.1016/j.tvjl.2017.01.006 28190503

[23] H. Hermans , R. A. Lloyd‐Edwards , A. J. Ferrão‐van Sommeren , et al., “Comparing Blind and Ultrasound‐Guided Retrobulbar Nerve Blocks in Equine Cadavers: The Training Effect,” Animals: An Open Access Journal From MDPI 12, no. 2 (2022): 154. 10.3390/ani12020154 PMC8772549 35049776

[24] A. E. Zibura , J. H. Salmon , B. Belda Lopez , B. D. X. Lascelles , and H. D. Westermeyer , “Glaucoma‐Associated Pain Results in Mechanical Sensitivity Changes in Dogs: A Pilot Study,” Veterinary Ophthalmology 24 (2021): 116–124. 10.1111/vop.12800 32608141

[25] V. Rabbogliatti , D. De Zani , D. D. Zani , et al., “Comparison of Four Peribulbar Anaesthetic Techniques: A Preliminary Study in Equine Cadavers,” Veterinary Anaesthesia and Analgesia 48, no. 3 (2021): 442–450. 10.1016/j.vaa.2020.10.009 33640273

[26] S. Stadler , M. Dennler , U. Hetzel , et al., “Sub‐Tenon's Injection in Equine Cadaver Eyes: MRI Visualization of Anesthetic Fluid Distribution and Comparison of Two Different Volumes,” Veterinary Ophthalmology 20, no. 6 (2017): 488–495. 10.1111/vop.12452 28008696

[27] O. Kiesse , P. Torgerson , S. Pot , et al., “Sub‐Tenon's Anesthesia in Equine Cataract Surgery and Vitrectomy: A Retrospective Case Series (2018–2022),” Schweizer Archiv Fur Tierheilkunde 166, no. 11 (2024): 563–572. 10.17236/sat00435 39470436

