# Supplementary material for: Feasibility of Enucleation in Standing Sedated Horses With Retrobulbar Anesthesia and Auriculopalpebral Nerve Block: Impact of Ultrasound Guidance in a Case Series
Source: Vet Ophthalmol. 2026 Oct 27;29(6):e70282. doi: 10.1111/vop.70282 (PMC13713594; doi:10.1111/vop.70282)
Supplement: Supplementary file 1 [file VOP-29-0-s001.docx]

**Supplementary Information 1:** Postoperative pain scoring system, applied at 2 and 24 hours following enucleation performed under standing sedation in a prospective clinical study (EQUUS-FAP Equine Utrecht University Scale for Facial Assessment of Pain as described by van Loon et al.). Due to omission of one criterion (*italics*), the maximum achievable score in this study was 16 points rather than 18.

| **Data** | **Categories** | **Score** |
| --- | --- | --- |
| Head | Normal head movement/interested in environment | 0 |
|  | Less movement | 1 |
|  | No movement | 2 |
| *Eyelids* | *Opened, sclera can be seen in case of eye/head movement* | *0* |
|  | *More opened eyes or tightening of eyelids. An edge of the sclera can be seen 50% of the time* | *1* |
|  | *Obviously more opened eyes or obvious tightening of eyelids. Sclera can be seen >50% of the time* | *2* |
| Focus | Focussed on environment | 0 |
|  | Less focussed on environment | 1 |
|  | Not focussed on environment | 2 |
| Nostrils | Relaxed | 0 |
|  | A bit more opened | 1 |
|  | Obviously more opened, nostril flaring and possibly audible breathing | 2 |
| Corners mouth/lips | Relaxed | 0 |
|  | Lifted slightly | 1 |
|  | Obviously lifted | 2 |
| Muscle tone head | No fasciculations | 0 |
|  | Mild fasciculations | 1 |
|  | Obvious fasciculations | 2 |
| Flehming and/or yawning | Not seen | 0 |
|  | Seen | 2 |
| Teeth grinding and/or moaning | Not heard | 0 |
|  | Heard | 2 |
| Ears | Position: Orientation towards sound/clear response with both ears or ear closest to source | 0 |
|  | Delayed/reduced response to sounds | 1 |
|  | Position: backwards/no response to sounds | 2 |
| **Total** |  | **…/16** |
